# Retention strategies in longitudinal cohort studies: a systematic review and meta-analysis

**DOI:** 10.1186/s12874-018-0586-7

**Published:** 2018-11-26

**Authors:** Samantha Teague, George J. Youssef, Jacqui A. Macdonald, Emma Sciberras, Adrian Shatte, Matthew Fuller-Tyszkiewicz, Chris Greenwood, Jennifer McIntosh, Craig A. Olsson, Delyse Hutchinson, the SEED Lifecourse Sciences Theme, Sharyn Bant, Sophie Barker, Anna Booth, Tanja Capic, Laura Di Manno, Alisha Gulenc, Genevieve Le Bas, Primrose Letcher, Claire Ann Lubotzky, Jessica Opie, Melissa O’Shea, Evelyn Tan, Jo Williams

**Affiliations:** 1 Centre for Social and Early Emotional Development, School of Psychology, Faculty of Health, Deakin University, Burwood, Geelong, Victoria 3125 Australia; 2 Murdoch Children’s Research Institute, Centre for Adolescent Health, Royal Children’s Hospital, Melbourne, Australia; 3 Department of Paediatrics, Royal Children’s Hospital, University of Melbourne, Melbourne, Australia; 4 Melbourne School of Psychological Sciences, University of Melbourne, Parkville, Australia; 5 National Drug and Alcohol Research Centre, University of New South Wales, Sydney, Australia; 6 School of Engineering & Information Technology, Faculty of Science & Technology, Federation University, Melbourne, Australia

**Keywords:** Retention, Attrition, Cohort, Longitudinal, Engagement, Follow-up, Drop-out

## Abstract

**Background:**

Participant retention strategies that minimise attrition in longitudinal cohort studies have evolved considerably in recent years. This study aimed to assess, via systematic review and meta-analysis, the effectiveness of both traditional strategies and contemporary innovations for retention adopted by longitudinal cohort studies in the past decade.

**Methods:**

Health research databases were searched for retention strategies used within longitudinal cohort studies published in the 10-years prior, with 143 eligible longitudinal cohort studies identified (141 articles; sample size range: 30 to 61,895). Details on retention strategies and rates, research designs, and participant demographics were extracted. Meta-analyses of retained proportions were performed to examine the association between cohort retention rate and individual and thematically grouped retention strategies.

**Results:**

Results identified 95 retention strategies, broadly classed as either: *barrier-reduction, community-building, follow-up/reminder,* or *tracing* strategies. Forty-four of these strategies had not been identified in previous reviews. Meta-regressions indicated that studies using barrier-reduction strategies retained 10% more of their sample (95%CI [0.13 to 1.08]; *p* = .01); however, studies using follow-up/reminder strategies lost an additional 10% of their sample (95%CI [− 1.19 to − 0.21]; *p* = .02). The overall number of strategies employed was not associated with retention.

**Conclusions:**

Employing a larger number of retention strategies may not be associated with improved retention in longitudinal cohort studies, contrary to earlier narrative reviews. Results suggest that strategies that aim to reduce participant burden (e.g., flexibility in data collection methods) might be most effective in maximising cohort retention.

**Electronic supplementary material:**

The online version of this article (10.1186/s12874-018-0586-7) contains supplementary material, which is available to authorized users.

## Background

Longitudinal cohort studies play a central role in advancing understanding of the onset and progression of physical and mental health problems. Cohort studies assess, and often compare, the incidence of a condition within a group of people who share common characteristics (e.g., being born in the same year) [1]. A key advantage of longitudinal cohort studies over other research designs is that repeated measures data temporally orders exposures and outcomes to facilitate causal inference [2]. However, significant and systematic attrition can reduce the generalisability of outcomes and the statistical power to detect effects of interest [3]. Systematic attrition in longitudinal research occurs most often in older, non-white male participants with limited education and/or multiple health problems [4]. Long duration and repeated assessments can also increase attrition due to the significant burden on participants [4]. Given the expense of longitudinal cohort studies, effective strategies that engage and retain cohort participants are critical to the integrity of research outcomes [5, 6].

In the last decade, longitudinal data collection methods and cohort retention strategies have evolved considerably. So too have participant expectations of organisations (research and otherwise) that seek information from individuals [7, 8]. Established retention strategies within longitudinal cohort studies include: cash or gift incentives, sending reminder letters to participants, re-sending surveys, and offering alternative methods of data collection (for a review, see [6]). Booker et al. [6] demonstrated that these strategies were effective in longitudinal cohort studies that used the traditional data collection methods of postal surveys, face-to-face visits (home or on-site), and telephone interviews or surveys. However, these cohort retention strategies may not be as well suited to contemporary methods of collecting longitudinal data, such as web and mobile surveys [9], wearable sensors (e.g., FitBits) [10], short message services (SMS) [11], and groupware systems (e.g., video conferencing) [12]. Novel methods of engaging participants such as web advertising [13], social media [14], and electronic reminders [15], are also now being employed in cohort studies using both traditional and modern longitudinal data collection methods.

A systematic review on the effectiveness of established and emerging cohort retention strategies in longitudinal cohort studies would provide guidance to researchers and funders on maximising cohort maintenance within these high investment programs of research. Previous reviews of retention strategies in health research include [4, 6, 16, 17]; only one of these reviews focused specifically on longitudinal cohort research designs [6]. Booker et al. [6] conducted a narrative review of retention strategies in longitudinal cohort studies, including incentives, reminders, repeat visits/questionnaires, and alternative methods of data collection, finding that incentives and reminder strategies improved cohort retention. However, this review was limited by the small number of studies identified for each retention strategy, which resulted in the identification of a restricted breadth of retention strategies and the inability to synthesise findings empirically.

Further, Booker et al. [6] did not include research completed after 2006 and thus were unable to investigate emerging cohort retention strategies. Brueton et al. [16] completed a more recent review of retention strategies that included both established and emerging digital data collection retention strategies. However, the authors specifically excluded longitudinal cohort studies and instead focused on participant retention in intervention trials. Differences between intervention and longitudinal cohort studies, such as research design factors (e.g., study duration) and the motivations of the participants in joining or withdrawing from studies, may impact the usefulness of retention strategies across both study designs [4, 6].

A review of retention strategies reported in modern longitudinal cohort studies is pertinent and timely, given the emergence of digital retention strategies alongside established retention methods. Maximising cohort retention in longitudinal research can reduce the administration costs of conducting research, improve the efficiency of research processes, and reduce outcome biases for studies by adopting an evidence-based cohort retention framework. In this review, we aimed to: (i) identify retention strategies used in recent longitudinal cohort studies; (ii) examine whether retention rate was moderated by different study or participant characteristics (i.e., number of waves, study duration, sample size, population type, gender, age, country); (iii) estimate the retention rate in studies that use specific retention strategies, and contrast this retention rate with studies that do not use specific retention strategies; (iv) examine whether retention rate is associated with the number of retention strategies used; (v) examine which retention strategies were the strongest independent predictors of retention rate; and (vi) contrast the retention rate based on whether studies utilised emerging or established strategies. Moreover, to ensure that recent innovations in retention strategies were identified, this review focused on literature published within the past 10 years.

## Method

### Search strategy

A systematic review was performed as per the Preferred Reporting Items for Systematic Reviews and Meta-Analyses (PRISMA) guidelines [18]. Two search strategies were implemented. First, the electronic databases Medline, PsycINFO, Embase, CINAHL, AMED, and the Cochrane Library, were searched in July, 2016 using search terms relevant to three themes: (i) attrition, (ii) retention, and (iii) study design (Additional file 1: Table S1). The electronic search was limited to articles published from 2006 onwards in English, to avoid duplication of literature with the previous review on this topic [6]. The search was adapted to suit each database. Second, the reference lists of all articles selected for review were manually searched.

### Inclusion and exclusion criteria

The inclusion and exclusion criteria were determined prior to implementing the search strategy. Articles were included in the review if: (i) the article described a cohort study, which was defined as a representative sample of a group or population who share a common experience or condition [2], (ii) the article reported at least one wave of follow-up data collection with a participant/proxy, (iii) participant retention data were reported, and (iv) retention strategies were reported. Articles were excluded if: (i) the article was not available in English; (ii) the article was not published in a peer-reviewed publication (e.g., conference abstracts or dissertations); and, (iii) the article’s research design was cross-sectional, involved data linkage only, or the article was a clinical or non-clinical trial evaluating the effectiveness of treatment regimens or intervention/prevention programmes (for an existing review of retention in intervention studies, see (12)).

### Study selection, data extraction, and quality assessment

**Fig. 1:**
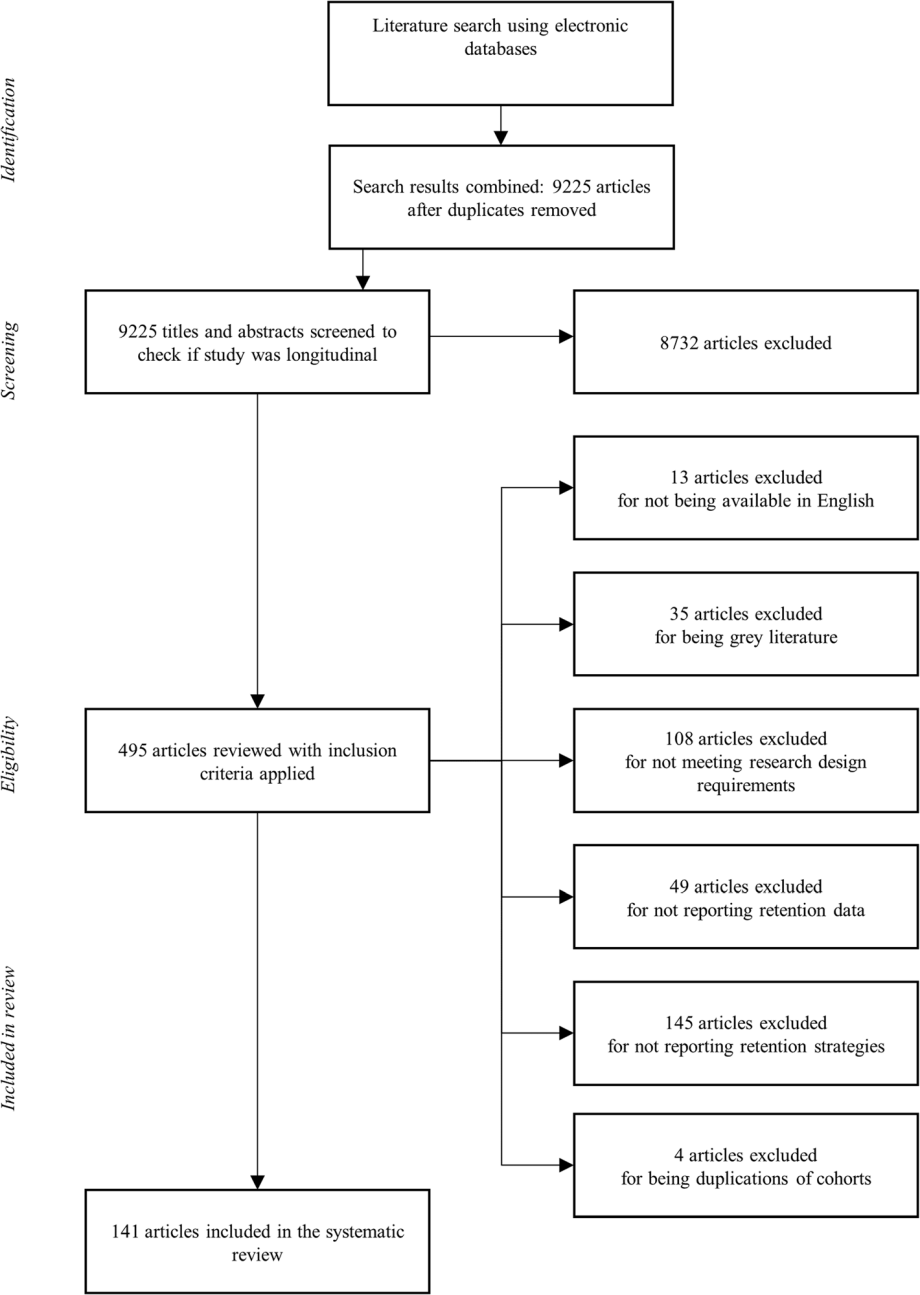
PRISMA procedural flow chart of the search and identification process

The search strategy resulted in 9225 articles after removing duplicates. In total, 141 articles were identified, screened, and determined to be eligible for inclusion (see Fig. 1). Data were extracted and summarised for each of the 141 articles on: (i) the research design, including baseline sample population and sample size, the number of data collection waves reported, and the duration in years between the first and last waves of data collection; (ii) the cohort demographics, including mean sample age at baseline (or age range if mean age was not reported), proportion of male participants, country of cohort participants, and whether the cohort was clinical or non-clinical; and (iii) retention data, including the retention rate between baseline and the final data collection wave reported, and the specific retention strategies. Finally, we examined the suitability of each article in addressing the current study’s research question. Articles that listed cohort attrition or retention as a research question or objective were categorised as “retention-focused”, and conversely articles that did not focus on attrition or retention were categorised as “non-retention-focused”. No articles were excluded on the basis of this quality assessment.

### Statistical method

We used meta-analysis (and meta-regression) to address the aims of the study. Meta-analyses were conducted using the Metafor package v1.9.8 [19] in R software v3.3.1 [20]. The retention rate, defined as the number of individuals who remained in the study at the last wave of data collection as a proportion of the total number of participants recruited at the baseline assessment, was the primary effect size measure of interest. All meta-analyses were conducted using inverse variance weighting, with random effects specified to account for between study heterogeneity. A binomial-normal model (with logit link) was used as the basis for analysis, which is appropriate when the effect size of interest is measured as a proportion. Where appropriate, meta-analytic effects were back-transformed to represent the median meta-analytic retention rate. We also report the I^2^ statistic as a measure of study heterogeneity, interpreted using the guidelines of Higgins et al. [21]. Meta-analyses were conducted when at least two independent studies contributed to the meta-analysis.

To examine the effect of gender on retention rate, we created a binary variable to denote studies as comprising a higher proportion of either male or female samples (Proportion of male participants in “male” grouping: *M(SD)* = 73.6%(0.20); Proportion of female participants in “female” grouping: *M(SD)* = 75.0%(0.21)). To examine the effect of country development level on retention rate, each study country was categorised as either high or low development level by using a mean-split of each nation’s Human Development Index – a measure of relative opportunity for longevity, education, and income, with a score range of 0 (low) to 1 (high) (Low HDI group *M(SD)* = 0.66(0.10); High HDI group *M(SD)* = 0.92(0.02)) [22]. Retention strategies were coded as either *established* or *emerging*, depending on their presence or absence in any of the earlier systematic reviews on participant retention strategies [4, 6, 16, 17]. Finally, all meta-regressions adjusted for study duration and number of waves (except when these were specifically examined as predictor variables), given these were deemed to be likely confounding variables in analyses.

## Results

### Cohort, participant, and article characteristics

The 141 articles identified for review described 143 cohorts (41 clinical and 102 non-clinical). Cohorts are summarised in Table 1. Overall the mean sample size reported in the first wave of each article was 3585 participants (range = 30 to 61,895). Articles reported a mean retention rate of 73.5% (SD = 20.1%), with 4.6 waves (SD = 8.0), over 4.3 years (SD = 5.0). The average baseline participant age was 30.0 years (SD = 22.0), and the average baseline proportion of male participants across samples was 40% (SD = 0.30). Studies were conducted in 28 different countries with a mean Human Development Index of 0.79 (SD = 0.15), indicating that studies were more likely to be conducted in countries with high-levels human development. Cohort attrition/retention was identified as a specific research question or objective of interest in 55 of the 141 articles, indicating that most articles were not focused on participant retention. Retention-focused articles reported significantly more retention strategies than non-retention-focused articles (non-retention-focused: *M(SD)* = 3.3(3.1); retention-focused: *M(SD)* = 11.0(7.02); *t*_(141)_ = − 9.00, *p* < .001); however, no differences were found for the study sample size, number of waves, study duration, or retention rate. High heterogeneity was identified in all results, as expected given the diversity of research questions, methodologies, and cohorts across articles [21].

**Table 1:** Description of cohorts reported in the included articles

Sample	Reference	Study Name	Wave 1 Sample Size	Wave 1 Mean Age	Overall Retention Rate	No. Waves	Study Duration (years)	No. Retention Strategies
Clinical cohort studies								
Adolescent and adult non-injecting heroin users	[32]	Project Brown	300	16–40	98%	2	1.00	9
Adolescent/young adult cancer patients	[33]	Resilience in Adolescents and Young Adults with Cancer Study	52	17.6	35%	3	1.50	4
Adolescent/Young Adult mobile young injection drug users	[34]		101	22	48%	6	2.00	9
Adolescents and young adults with Type 1 Diabetes	[35]	Young Adult Diabetes Assessment (YADA)	204	17–18	97%	3	5.00	18
Adult asthmatic pregnant women	[36]	Syracuse AUDIT (Assessment of Urban Dwellings for Indoor Toxics)	103	25.4	86%	5	1.00	9
Adult cannabis users	[37]		193	32	84%	90	0.25	3
Adult entitlement claimants from the Accident Compensation Corporation	[38]	Prospective Outcomes of Injury Study (POIS)	2856	18–64	79%	4	2.00	4
Adult major trauma patients	[39]	Victorian State Trauma Registry	1102	40	70%	2	0.50	3
Adult myocardial infarction survivors	[40]	Western New York Acute Myocardial Infarction (MI) Study	884	54	90%	4	7.00	2
Adult parents of overweight children with low-income	[41]		37	20–50+	46%	26	1.00	14
Adult Puerto Rican/Mexicans with a mental health diagnosis	[42]		68	18–50	59%	3	5.00	20
Adult smokers and non-smoker comparisons	[43]	International Tobacco Control (ITC) China Survey	6001	18–55+	68%	3	3.00	7
Adult spinal surgery patients	[44]	Danish spine surgery registry (Danespine)	506	58.94	100%	3	1.00	1
Adult survivors of ARDS	[45]	Toronto Acute Respiratory Distress Syndrome (ARDS) Study	109	–	86%	3	5.00	18
Adult survivors of SARS	[45]	Toronto Severe acute respiratory syndrome (SARS) Study	117	–	91%	2	2.00	17
Adults with diabetes	[46]	Living with Diabetes Study	3951	61.4	81%	3	3.00	15
Adult women at-risk of cardiovascular events	[47]	PREDICT Study	1110	21+	90%	9	2.00	12
Adult women at-risk of HIV infection	[48]		411	21	94%	2	1.00	4
Adult women breast cancer survivors	[49]		121	59.7	96%	2	1.00	3
Adult women with HIV/AIDS	[50]	Instituto de Pesquisa Clínica Evandro Chagas (IPEC) Cohort of Women Living with HIV/ AIDS followed up in Fundação Oswaldo Cruz (FIOCRUZ) Rio de Janeiro	225	32	56%	3	3.00	2
Adults at first-episode psychosis	[51]		71	18–60	70%	3	5.00	1
Adults at-risk for HIV infection	[52]		2191	18–49	77%	2	1.00	4
Adults at-risk of problem gambling plus comparison group	[53]	Quinte Longitudinal Study	4121	46.1	94%	5	5.00	1
Adults who self-harm	[54]		150	28.4	95%	3	6.00	4
Adults who use urinary catheters	[55]		33	43	100%	4	0.50	2
Adults with acute transient ischemic attack or stroke	[56]	Oxford Vascular Study	1236	75.2	98%	7	10.00	3
Adults with Alzheimers Disease	[57]	REAL.FR study	686	77.9	59%	2	2.00	3
Adults with Alzheimers Disease (AD) and their carers	[58]		40	78	81%	5	1.00	16
Adults with Alzheimers Disease or Mild Cognitive Impairment and comparison	[59]	Australian Imaging, Biomarkers and Lifestyle Flagship Study of Ageing (AIBL)	1112	69.7	90%	2	1.50	3
Adults with aneurysmal subarachnoid hemorrhage (aSAH)	[60]	Family Caregiver study	59	52	83%	4	1.00	5
Adults with back pain	[61]		250	30–59	68%	14	7.00	3
Adults with primary malignant brain tumour (PMBT) and their caregivers	[60]	20-Hete Study	496	53.12	90%	3	1.00	3
Adults with primary Sjögren’s syndrome	[62]		222	52.5	70%	2	7.60	2
Adults with schizophrenia and comparison group	[63]		56	21	89%	2	2.00	1
Adults with Severe Traumatic Brain Injury	[64]	PariS-TBI study	504	42	60%	2	4.00	6
Adults with temporomandibular disorders	[65]	Orofacial Pain: Prospective Evaluation and Risk Assessment (OPPERA) Study	3263	31	84%	11	2.80	5
Adults with traumatic brain injury	[66]	Tasmanian Neurotrauma Register (TNTR)	947	36.1	19%	7	3.00	5
Adults with Traumatic Brain Injury	[67]		77	67.1	57%	3	0.50	1
Adult burn victims	[68]	Burns Registry of Australia and New Zealand	463	41.8	21%	5	2.00	4
Caregivers of adult cancer patients	[69]		206	57	85%	3	1.10	1
Child twins and their siblings	[70]	Australian Twin ADHD Project (ATAP)	1938	4–12	43%	3	9.00	3
Children at-risk of HIV infection	[71]	AIDS-ill families study	3515	13.5	97%	2	1.00	9
Children at-risk of thyroid cancer and comparison group	[72]		600	11	88%	2	8.00	9
Children exposed to Cocaine/opiate and comparison	[73]	Maternal Lifestyle Study (MLS)	13,888	0.1	76%	5	15.00	16
Children perinatally infected with HIV and comparison	[74]	IMPAACT P1055 Psychiatric Co-Morbidity Study	582	12.4	81%	2	2.00	3
Children who were former child soldiers	[75]		260	10–17	69%	3	6.00	3
Children with ADHD and a sibling for comparison	[26]	International Multicenter ADHD Genetics (IMAGE) study	459	11.4	76%	2	6.00	1
Children with ADHD and comparisons	[76]	Berkeley Girls with ADHD Longitudinal Study (BGALS)	228	9.6	95%	3	10.00	2
Female adolescent/young adult survivors of a mass campus shooting	[77]		812	19	81%	7	2.50	1
Infants at-risk of developing diabetes	[78]	The Environmental Determinants of Diabetets in the Young (TEDDY) study	4138	0.4	74%	3	1.00	1
Male sex workers	[79]		50	17–26+	34%	2	0.50	4
Men who have Sex with Men	[25]	Bangkok Men who have Sex with Men Cohort Study (BMCS)	1744	26	90%	10	3.00	3
Men who have Sex with Men	[80]		2607	22.7	22%	2	0.25	2
Men who have Sex with Men	[81]		710	18–54	74%	2	1.00	5
Men who have Sex with Men	[82]		1003	28	70%	8	2.60	1
Men who have Sex with Men	[83]		511	29	55%	3	0.75	5
Men who have Sex with Men	[84]		278	32	16%	3	1.00	5
Men who have Sex with Men	[85]		327	30.8	92%	3	1.00	1
Population at-risk for HIV infection	[86]		1000	13–49	77%	5	2.50	3
Non-clinical cohort studies								
Adolescent mother-child dyads	[87]		97	14–20	38%	3	4.00	9
Adolescent population	[88]	Danish Youth Cohort	12,498	13.4	25%	3	2.00	1
Adolescent population	[89]	Dating It Safe	964	16.1	86%	2	1.00	1
Adolescent population	[90]	Healthy Teens Longitudinal Study	611	14.8	66%	7	6.00	1
Adolescent population	[91]	International Youth Development Study (IYDS)	1858	13	98%	3	2.00	4
Adolescent population	[92]	TRacking Adolescents’ Individual Lives Survey (TRAILS)	2773	11.1	79%	4	8.00	1
Adolescent population	[93]	Youth Asset Study (YAS)	1117	12–17	97%	5	4.00	32
Adolescent population	[94]		1535	14.9	57%	2	1.00	1
Adolescent population	[95]		497	13.03	86%	6	6.00	1
Adolescent/Young adult twins	[96]	Minnesota Twin Family Study (MTFS)	1252	17	93%	4	12.00	1
Adult African American population	[97]	Religion and Health in African Americans (RHIAA) study	2803	54.86	40%	2	2.50	16
Adult African American women	[98]	Study of Environment, Lifestyle and Fibroids (SELF)	1696	23–34	87%	2	1.67	3
Adult Alaska Native and American Indian population	[99]	Education and Research Towards Health (EARTH) study	3828	18–55+	88%	2	1.50	18
Adult low income mothers	[100]	Welfare Client Longitudinal Study (WCLS)	498	18–35+	89%	2	1.00	11
Adult male population	[101]	Florey Adelaide Male Ageing Study (FAMAS)	1195	55	96%	2	1.00	14
Adult mother-child dyads	[102]		4318	0.2	84%	5	1.00	3
Adult mother-child dyads	[103]		365	13.7	64%	2	1.00	4
Adult officeworkers	[104]		53	42	100%	26	1.00	1
Adult online panel members	[105]	ATTEMPT Cohort	2009	47.9	52%	5	1.00	3
Adult online panel members	[106]		202	33.8	47%	3	0.00	1
Adult population	[107]	Baltimore Epidemiologic Catchment Area Follow-up	3481	18–65+	53%	3	23.00	1
Adult population	[108]	Healthy Aging in Neighborhoods of Diversity across the Life Span (HANDLS) study	3722	30–64	79%	3	4.00	12
Adult population	[109]	Heart Strategies Concentrating on Risk Evaluation (Heart SCORE) study	1841	59.1	84%	5	4.00	11
Adult population	[110]	Helsinki Aging Study (HAS)	170	80	42%	2	5.00	3
Adult population	[111]	Knee Clinical Assessment Study (CAS(K))	819	50–80+	95%	2	1.50	3
Adult population	[112]	Longitudinal Assessment of Women (LAW)	511	64.7	96%	5	5.00	16
Adult population	[113]	Midlife in the United States (MIDUS)	7108	25–74	75%	2	10.00	4
Adult population	[114]	MRC National Survey of Health and Development (NSHD)	3163	60–64	84%	2	9.00	2
Adult population	[115]	Netherlands Mental Health Survey and Incidence Study (NEMESIS-2)	18–64	18+	80%	2	3.00	9
Adult population	[116]	Netherlands Study of Depression and Anxiety (NESDA)	2981	39.9	87%	2	2.00	10
Adult population	[117]	New Zealand Attitudes and Values Study	6518	48	62%	4	3.00	12
Adult population	[118]	NutriNet-Santé Cohort Study	15,000	18+	44%	2	2.00	8
Adult population	[119]	People’s Republic of China-United States of America (PRC-USA) Collaborative Study of Cardiovascular and Cardiopulmonary Epidemiology	1739	57.7	94%	3	5.00	1
Adult population	[120]	Quinte Longitudinal Study (QLS)	4121	18–65+	94%	5	5.00	1
Adult population	[121]	Study of health in Pomerania (SHIP)	6267	20–79	84%	2	5.00	10
Adult population	[122]	Study of Use of Products and Exposure-Related Behavior (SUPERB)	481	36	47%	9	3.00	3
Adult population	[123]		700	48.8	71%	4	2.00	2
Adult pregnant women	[124]	Drakenstein Child Health Study (DCHS)	585	26.6	90%	2	1.33	6
Adult pregnant women	[125]	G-GrippeNet (GGNET) Project	153	34	78%	10	0.20	2
Adult pregnant women	[126]	Maternal Anxiety in Relation to Infant Development (MARI) Study	306	28	90%	7	2.00	2
Adult pregnant women	[127]	Mater-University Study of Pregnancy (MUSP)	6753	24.3	88%	6	27.00	5
Adult pregnant women	[128]	Pregnancy, Infection, and Nutrition Study	262	30	70%	2	2.00	5
Adult pregnant women	[129]		118	31.6	72%	4	1.00	1
Adult pregnant women	[130]		40,333	30.3	65%	2	0.75	1
Adult pregnant women	[131]		1040	18–34+	71%	3	1.10	1
Adult premenopausal women	[132]	Uterine Fibroid Study (UFS)	1141	35–49	85%	3	8.00	5
Adult South Asians living in US	[133]	Mediators of Atherosclerosis in South Asians Living in America (MASALA) study	906	40–84	48%	2	0.75	6
Adult veterans	[134]		1319	33	79%	2	1.00	4
Adult women	[135]	Australian Longitudinal Study on Women’s Health	14,247	18–23	77%	4	4.00	8
Adult women	[136]	Australian Longitudinal Study on Women’s Health	40,395	18–75	80%	2	6.00	5
Adult women	[137]	Manitoba Breast Screening Program	47,637	50–68	80%	2	2.50	5
Adult women	[138]		1435	40–50	72%	3	3.00	13
Adult women	[139]		48,125	38	91%	2	12.00	5
Adult women hoping to become pregnant	[140]		30	29.4	43%	4	1.50	2
Adult/Young Adult Probationers	[141]		199	17–35	52%	5	15.00	7
Birth cohort	[142]	Australian Aboriginal Birth Cohort study	686	0	72%	3	18.00	31
Birth cohort	[143]	Birth to Twenty (BT20) birth cohort	3273	0	70%	19	16.00	16
Birth cohort	[144]	Danish National Birth Cohort	61,895	0	63%	2	7.00	1
Birth cohort	[145]	ECAGE Project (Study of Food Intake and Eating Behavior of Pregnant Women)	462	0	94%	3	0.65	2
Birth cohort	[146]	Environments for Healthy Living (EHL)	3368	0	65%	2	5.50	7
Birth cohort	[147]	Geographic research on wellbeing (GROW) study	9256	7	33%	2	7.00	9
Birth cohort	[148]	Growing up in New Zealand	6846	0	95%	2	0.75	23
Birth cohort	[149]	Japan Children’s Study (JCS)	467	0.3	81%	6	3.50	13
Birth cohort	[150]	Nascita e INFanzia gli Effetti dell’Ambiente (NINFEA) cohort	7003	0	78%	4	4.00	6
Birth cohort	[151]		413	0	95%	2	0.50	1
Birth cohort	[152]		1196	0	46%	5	30.00	1
Birth cohort of children from Lesbian parents	[153]	US National Longitudinal Lesbian Family Study (NLLFS)	154	0	93%	5	17.00	1
Child African-American population and their parents	[154]		76	3.4	70%	2	3.50	18
Child monozygotic (MZ) and dizygotic (DZ) twins	[155]	University of Southern California Study of Risk Factors for Antisocial Behavior (USC RFAB)	1569	9	59%	5	8.00	10
Child population	[156]	Danish youth cohort Vestliv	3054	14.5	64%	3	6.00	1
Child population	[157]	Ho Chi Minh City Youth Cohort	759	11.8	77%	5	5.00	4
Child population	[158]		405	11	91%	4	4.00	17
Indigenous adolescents	[159]		671	11.3	79%	8	8.00	7
Mother-child dyads	[160]	Center for Oral Health Research in Appalachia 2 (COHRA2) Study	744	28.4	79%	2	2.50	1
Older adults	[161]	Cardiovascular Health Study (CHS)	5888	73	46%	2	7.00	2
Older adults	[162]	Chinese Longitudinal Healthy Longevity Survey (CLHLS)	16,020	65+	56%	3	2.00	1
Older adults	[163]	Longitudinal Aging Study Amsterdam (LASA)	3107	70	32%	6	17.00	6
Older adults	[164]	New England Centenarian Study (NECS)	759	97+	86%	2	3.50	1
Older adults	[165]	Newcastle 85+ Study	854	85+	40%	4	5.00	11
Older adults	[166]	Physiological Research to Improve Sleep and Memory Project	78.2	70+	83%	3	2.00	24
Older adults	[167]	UAB Study of Aging	1000	65+	95%	2	4.00	16
Population during political turmoil	[168]		889	36	89%	2	0.50	1
Population during political turmoil	[169]		1022	33.9	85%	2	6.00	7
Young adult women population	[170]	Chlamydia Incidence and Re-infection Rates Study (CIRIS)	1116	21	79%	3	1.00	13
Overall Mean (Std Dev)			3459 (8979)	24.7 (23.5)	73.9% (20.1%)	4.6 (8.0)	4.5 (5.1)	6.2 (6.2)

### Relationship between retention rate and study or participant characteristics

To examine whether retention rate was moderated by study characteristics (i.e., number of waves, study duration, sample size, study focus on retention strategies or not) or by participant characteristics (i.e., population type, gender, age, country development level), a series of meta-regressions was performed, one for each characteristic under examination. Retention rate was not moderated by: number of waves (b < 0.001; 95%CI [− 0.02 to 0.03], *p* = .77); study duration (b = − 0.02; 95%CI [− 0.06 to 0.02]; *p* = .34); sample size (b < − 0.001; 95%CI [− 0.00 to 0.00]; *p* = 0.48); or articles’ focus on retention strategies (b = − 0.12; 95%CI [− 0.54 to 0.30]; *p* = .57). Additionally, retention rate was not associated with the sample characteristics of: cohort type (clinical or non-clinical) (b = 0.04; 95%CI [− 0.42 to – 0.51]; *p* = .86); mean age (b = 0.02; 95%CI [− 0.01 to 0.01]; *p* = .74); or country development level (b = 0.11; 95%CI [− 0.46 to 0.68]; *p* = .71). However, gender was a significant moderator of retention rate (b = − 0.67; 95%CI [− 1.14 to − 0.20]; *p* < .01)). Namely, cohorts with more female participants (median retention = 81.5%, 95%CI [77.6% to 84.9%]) reported higher retention rates than articles with more male participants (median retention = 70.1%; 95%CI [60.1% to 78.5%]), after controlling for study duration and number of waves.

### Relationship between retention rate and retention strategy types

A total of 95 retention strategies was identified, with an average of 6.2 strategies per article (SD = 6.2). The most common retention strategies were: cash/voucher incentives to complete a follow-up assessment (*n* = 59), sending a postcard or letter reminder to complete a follow-up assessment (*n* = 43), and offering participants alternative methods of data collection, such as completing an interview face-to-face or over the phone (*n* = 36).

Retention strategies were grouped into four main retention strategy domains: (i) *barrier-reduction strategies*, such as offering childcare services, assistance with parking and transport, and engaging a participant sub-sample to evaluate data collection methods for the next wave; (ii) *community-building strategies*, such as creating a recognisable study brand via logos and colour schemes, giving away study merchandise to create a sense of project community (e.g., t-shirts with study logo), and sharing study results, news and events with participants via newsletters, social media, and feedback reports; (iii) *strategies to improve follow-up rates within each wave*, including cash or voucher incentives for varying levels of assessment completion, and use of phone calls, SMS, house visits, mail and email reminders to participants to complete assessments; and (iv) *tracing strategies*, such as collecting the details of alternative contact persons for each participant at baseline, using public or non-public records to find updated contact information for participants, and collecting detailed participant contact information via a locator document (e.g. full name, address, social security number, phone numbers, email addresses, etc.). The most commonly reported category was strategies to improve follow-up rates within waves, identified 306 times within the 143 cohorts, followed by barrier-reduction strategies (adopted 268 times), community-building strategies (adopted 181 times), and tracing strategies (adopted 138 times).

Table 2 presents the retention strategies used, grouped by retention strategy domain. It compares the retention rate for those studies that did, or did not utilise a specific retention strategy type or domain. Of the 95 individual retention strategies examined, three demonstrated moderation of the retention rate. First, improved retention was associated with offering participants alternative methods of data collection (e.g., completing an interview face-to-face or over the phone) (median retention using strategy = 86.1%; median retention not using strategy = 76.3%; b = 0.24, *p* = .01), and having participants complete a locator document at baseline (median retention using strategy = 90.9%; median retention not using strategy = 78.1%; b = 0.49, *p* = 0.02). Finally, lower retention was associated with use of phone call reminders to participants to complete a follow-up wave (median retention using strategy = 72.7%; median retention not using strategy = 80.6%; b = 0.25, *p =* .05). There was weak evidence against the null hypothesis of no moderation effect for a further three strategies. This included having consistent research team members (median retention using strategy = 87.3%; median retention not using strategy = 78.1%; b = 0.67, *p* = .09); offering site and home visits for data collection (median retention using strategy = 83.9%; median retention not using strategy = 77.4%; b = 0.46, *p* = .07); and sending participants thank you, birthday or holiday cards (median retention using strategy = 84.9%; median retention not using strategy = 77.5%; b = 0.50, *p* = .07). There was no evidence to support a moderated retention rate by any other specific retention strategy type.

**Table 2:** Median meta-analytic retention rates for each retention strategy

	Studies using strategy		Studies not using strategy		Absolute Difference	*P*	I^2^
	*N*	Retention Rate (Lower CI - Upper CI)	*N*	Retention Rate (Lower CI - Upper CI)			
Reducing barriers to participation (Any vs None)	109	0.81 (0.77–0.84)	34	0.71 (0.62–0.78)	0.10	0.01*	99.87%
Adapt materials for mixed abilities (e.g., non-English speaking participants)	4	0.74 (0.37–0.93)	139	0.79 (0.75–0.82)	− 0.05	0.67	99.88%
Adjust inclusion criteria	1						na
Adjust lab to be more home-like, less clinical	2	0.81 (0.77–0.84)	141	0.79 (0.75–0.82)	0.02	0.84	99.89%
Advisory group	2	0.68 (0.58–0.77)	141	0.79 (0.75–0.82)	− 0.11	0.56	99.89%
Alternative method of data collection	36	0.86 (0.78–0.92)	107	0.76 (0.72–0.8)	0.10	0.01**	99.88%
Anonymity for participants	1						na
Assistance with postage costs	5	0.88 (0.73–0.95)	138	0.79 (0.75–0.82)	0.09	0.21	99.89%
Assistance with transport/parking/directions	12	0.8 (0.73–0.86)	131	0.79 (0.75–0.82)	0.01	0.72	99.88%
Catering/refreshments	10	0.87 (0.8–0.92)	133	0.78 (0.74–0.82)	0.09	0.13	99.88%
Child care	3	0.68 (0.51–0.82)	140	0.79 (0.75–0.82)	− 0.11	0.36	99.89%
Consistency in research staff	11	0.87 (0.77–0.93)	132	0.78 (0.74–0.82)	0.09	0.09	99.88%
Partial data collected from proxy/data linkage	27	0.81 (0.73–0.86)	116	0.79 (0.74–0.82)	0.02	0.42	99.88%
Adapt materials for different languages	12	0.84 (0.72–0.92)	131	0.78 (0.75–0.82)	0.06	0.39	99.88%
Extended data collection window	7	0.74 (0.54–0.88)	136	0.79 (0.75–0.82)	− 0.05	0.52	99.88%
Flexibility from research team (e.g., hours called, scheduling)	24	0.83 (0.76–0.89)	119	0.78 (0.74–0.82)	0.05	0.23	99.88%
Focus group on survey design	2	0.72 (0.7–0.75)	141	0.79 (0.75–0.82)	−0.07	0.93	99.89%
Hiring, training, and support of staff	21	0.84 (0.77–0.9)	122	0.78 (0.74–0.82)	0.06	0.11	99.88%
Matching staff to participants, e.g., by language spoken, nature of questions	2	0.94 (0.91–0.96)	141	0.79 (0.75–0.82)	0.15	0.14	99.88%
Minimising time between data collection points	1						na
Pilot testing	4	0.81 (0.63–0.91)	139	0.79 (0.75–0.82)	0.02	0.93	99.89%
Prioritising measures	12	0.73 (0.6–0.82)	131	0.8 (0.76–0.83)	−0.07	0.37	99.89%
Recruiting for long-term retention	10	0.83 (0.67–0.92)	133	0.79 (0.75–0.82)	0.04	0.50	99.87%
Schedule two participants simultaneously - often family or friends	2	0.76 (0.66–0.84)	141	0.79 (0.75–0.82)	−0.03	0.92	99.89%
Simple, efficient procedure	1						na
Site and home visits	31	0.84 (0.78–0.88)	112	0.77 (0.73–0.81)	0.07	0.07	99.88%
Skip waves	15	0.84 (0.75–0.9)	128	0.78 (0.74–0.82)	0.06	0.25	99.88%
Splitting data collection over multiple sessions	2	0.79 (0.78–0.81)	141	0.79 (0.75–0.82)	0.00	0.90	99.89%
Survey design (e.g., order of survey items)	3	0.77 (0.52–0.91)	140	0.79 (0.75–0.82)	−0.02	0.88	99.88%
Toll-free project phone number	5	0.75 (0.57–0.88)	138	0.79 (0.75–0.82)	−0.04	0.75	99.89%
Creating a project community (Any vs None)	59	0.80 (0.75–0.85)	84	0.78 (0.73–0.82)	0.02	0.48	99.88%
Advisory group	2	0.68 (0.58–0.77)	141	0.79 (0.75–0.82)	− 0.11	0.56	99.89%
Branding	14	0.79 (0.65–0.89)	129	0.79 (0.75–0.82)	0.00	0.99	99.88%
Certificate of appreciation/completion	2	0.83 (0.28–0.98)	141	0.79 (0.75–0.82)	0.04	0.82	99.88%
Champion participants	1						na
Educating the community on research	5	0.87 (0.7–0.95)	138	0.79 (0.75–0.82)	0.08	0.40	99.89%
Emphasising benefits of study	3	0.82 (0.7–0.9)	140	0.79 (0.75–0.82)	0.03	0.79	99.89%
Events/opportunity to meet other participants	9	0.69 (0.54–0.82)	134	0.8 (0.76–0.83)	−0.11	0.23	99.88%
Feedback report	10	0.84 (0.73–0.91)	133	0.79 (0.75–0.82)	0.05	0.39	99.88%
Gaining support of relevant institutions and organisations	4	0.85 (0.71–0.93)	139	0.79 (0.75–0.82)	0.06	0.57	99.89%
Gift/ freebies	19	0.8 (0.67–0.88)	124	0.79 (0.75–0.82)	0.01	0.90	99.89%
Hiring, training, and support of staff	21	0.84 (0.77–0.9)	122	0.78 (0.74–0.82)	0.06	0.11	99.88%
Letter from chief investigator	1						na
Media coverage	3	0.7 (0.69–0.72)	140	0.79 (0.75–0.82)	−0.09	0.82	99.89%
Newsletter/e-newsletter	24	0.83 (0.76–0.89)	119	0.78 (0.74–0.82)	0.05	0.23	99.88%
Opportunity to participate in other research	1						na
Photo album	2	0.72 (0.69–0.75)	141	0.79 (0.75–0.82)	−0.07	0.75	99.89%
Building rapport	22	0.79 (0.69–0.86)	121	0.79 (0.75–0.82)	0.00	0.97	99.89%
Sharing study results	5	0.88 (0.66–0.97)	138	0.79 (0.75–0.82)	0.09	0.24	99.89%
Social media	2	0.89 (0.72–0.96)	141	0.79 (0.75–0.82)	0.10	0.39	99.89%
Study membership card	1						na
Thank you, birthday, and holiday cards	25	0.85 (0.79–0.9)	118	0.78 (0.73–0.81)	0.07	0.07	99.88%
Time with chief investigator	2	0.92 (0.8–0.97)	141	0.79 (0.75–0.82)	0.13	0.24	99.89%
Website	3	0.80 (0.47–0.94)	140	0.79 (0.75–0.82)	0.01	1.00	99.88%
Follow-up/Reminder strategies (Any vs None)	111	0.76 (0.72–0.80)	32	0.86 (0.79–0.91)	−0.10	0.02*	99.86%
Follow-up brochure	2	0.78 (0.74–0.81)	141	0.79 (0.75–0.82)	− 0.01	0.97	99.89%
Budgeting for multiple contact attempts	1						na
Extra incentive to complete all data collection points	2	0.93 (0.77–0.98)	141	0.79 (0.75–0.82)	0.14	0.17	99.89%
Gift/ freebies incentives (e.g., t-shirts, discount cards)	18	0.8 (0.67–0.88)	125	0.79 (0.75–0.82)	0.01	0.90	99.89%
Hiring, training, and support of staff	21	0.84 (0.77–0.9)	122	0.78 (0.74–0.82)	0.06	0.11	99.88%
Incentive (cash/vouchers)	59	0.78 (0.72–0.82)	84	0.8 (0.75–0.84)	−0.02	0.45	99.88%
Incentive increasing value over time	10	0.78 (0.62–0.88)	133	0.79 (0.75–0.82)	− 0.01	0.81	99.88%
Incentives raffles/competitions	11	0.86 (0.71–0.94)	132	0.78 (0.75–0.82)	0.08	0.22	99.88%
Increased incentive for hard-to-reach Pp	6	0.68 (0.47–0.84)	137	0.79 (0.76–0.83)	−0.11	0.24	99.88%
Limiting number of calls etc. based on participants’ response	1						na
Medical assistance (e.g., diagnostic testing)	27	0.74 (0.64–0.82)	116	0.8 (0.76–0.84)	−0.06	0.17	99.88%
Phone Follow-up	11	0.80 (0.67–0.89)	132	0.79 (0.75–0.82)	0.01	0.90	99.88%
Provide referrals, e.g., medical or legal	9	0.85 (0.77–0.91)	134	0.78 (0.75–0.82)	0.07	0.26	99.89%
Resend survey once	6	0.77 (0.64–0.86)	137	0.79 (0.75–0.82)	−0.02	0.79	99.88%
Resend survey multiple times	10	0.76 (0.64–0.84)	133	0.79 (0.75–0.83)	−0.03	0.63	99.88%
SMS follow-up	1						na
Website follow-up	8	0.81 (0.62–0.91)	135	0.79 (0.75–0.82)	0.02	0.93	99.88%
Email reminder	13	0.73 (0.58–0.85)	130	0.79 (0.76–0.83)	−0.06	0.31	99.88%
Face-to-face reminder (e.g., home visit)	7	0.85 (0.67–0.94)	136	0.79 (0.75–0.82)	0.06	0.33	99.89%
Phone call reminder	34	0.73 (0.63–0.8)	109	0.81 (0.77–0.84)	−0.08	0.05*	99.88%
Postcard/letter reminder	43	0.77 (0.7–0.83)	100	0.80 (0.75–0.84)	−0.03	0.50	99.88%
SMS reminder	5	0.85 (0.8–0.9)	138	0.79 (0.75–0.82)	0.06	0.42	99.89%
Reminders (unspecified)	1						na
Tracing strategies (Any vs None)	53	0.80 (0.73–0.85)	90	0.78 (0.74–0.83)	0.02	0.62	99.88%
Tracing via alternative contacts	28	0.82 (0.75–0.87)	115	0.78 (0.74–0.82)	0.04	0.32	99.88%
Case-review meetings	1						na
Tracing via change of address cards	2	0.74 (0.43–0.91)	141	0.79 (0.75–0.82)	−0.05	0.95	99.89%
Tracing via email	2	0.74 (0.43–0.92)	141	0.79 (0.75–0.82)	−0.05	0.82	99.89%
Extensive location tracking information, e.g., known ‘hangouts’	1						na
Hiring, training, and support of staff	21	0.84 (0.77–0.9)	122	0.78 (0.74–0.82)	0.06	0.11	99.88%
Tracing via house visit	1						na
Tracing via incentive for staff members	2	0.72 (0.67–0.76)	141	0.79 (0.75–0.82)	−0.07	0.69	99.89%
Tracing via incentive to update contact details	3	0.86 (0.62–0.96)	140	0.79 (0.75–0.82)	0.07	0.43	99.88%
Tracing via letter	9	0.77 (0.51–0.91)	134	0.79 (0.75–0.82)	−0.02	0.72	99.89%
Tracing via locator form documentation*	7	0.91 (0.79–0.97)	136	0.78 (0.74–0.81)	0.13	0.02*	99.88%
Tracing via phone call	8	0.67 (0.51–0.8)	135	0.8 (0.76–0.83)	−0.13	0.12	99.88%
Tracing via private investigator	1						na
Tracing via SMS	1						na
Tracing via social media	3	0.79 (0.39–0.95)	140	0.79 (0.75–0.82)	0.00	0.92	99.89%
Tracing via tracing via public records	20	0.82 (0.73–0.88)	123	0.78 (0.74–0.82)	0.04	0.37	99.88%
Tracing via tracking database	15	0.83 (0.73–0.9)	128	0.78 (0.74–0.82)	0.05	0.32	99.88%
Tracing via update your details form	4	0.9 (0.81–0.96)	139	0.79 (0.75–0.82)	0.11	0.15	99.89%
Tracing via website	2	0.80 (0.79–0.81)	141	0.79 (0.75–0.82)	0.01	0.99	99.89%
Tracing via non-public records, e.g., apartment complex managers	7	0.82 (0.66–0.92)	136	0.79 (0.75–0.82)	0.03	0.59	99.89%

All inferential analyses adjusted for study duration and number of waves

*na* insufficient studies to perform meta-analysis

*N* No. effect in analysis

*p < .05

**p < .01

To examine whether the specific strategy domains of barrier-reduction, community-building, follow-up/reminder, and tracing retention strategies were associated with retention rate, a binary variable was created for each domain that denoted whether a study did or did not utilise one or more specific strategy types within that domain. As shown in Table 2, after controlling for study duration and number of waves, studies that utilised *any* barrier-reduction strategy had higher retention rates than those that did not use a barrier strategy (median retention using barrier strategies = 81.1%; median retention not using barrier strategies = 70.7%; b = 0.61, *p =* .01). Again after controlling for the study duration and number of waves, surprisingly, articles that reported use of at least one follow-up/reminder strategy had *lower* retention rates when compared to studies that did not utilise any follow-up/reminder (median retention using follow-up/reminder strategies = 76.4%; median retention not using follow-up/reminder strategies = 86.1%; b = − 0.32, *p* < .01). No relationships were found between retention rate and the use of any community-building or tracing retention strategies.

### Relationship between retention rate and number of strategies used

To examine whether the cumulative number of retention strategies was associated with retention rate, we meta-regressed retention rate on to continuous variables representing the cumulative number of strategies used across strategy domains, and then within each domain separately. Greater number of retention strategies used (across all domains) was not associated with higher retention rate (b = 0.02; 95%CI [− 0.12 to 0.05], *p* = .21). When examined within each domain, controlling for study duration and number of waves, we found accumulation of barrier-reduction strategies was associated with higher retention (b = 0.12; 95%CI [0.02 to 0.22]; *p =* .02). In separate meta-regressions, no relationships with retention were identified between number of community-building strategies (b = − 0.03; 95%CI [− 0.18 to 0.11]; *p =* 0.63), follow-up strategies (b = − 0.03; 95%CI [− 0.14 to 0.09]; *p* = 0.65), or tracing strategies (b = 0.10; 95%CI [− 0.07 to 0.28]; *p =* .25).

### Identifying strongest independent predictors of retention rate

Three separate meta-regression models were estimated to examine strongest predictors of retention rate within strategy domains and types. Table 3-Model 1 shows that when examining retention strategy types as cumulative variables for each domain, barrier-reduction was independently associated with higher retention (b = 0.17; 95%CI [0.03 to 0.31]; *p* = .02) and follow-up strategies was independently associated with lower retention (b = − 0.15; 95%CI [− 0.29 to − 0.01]; *p* = .04) beyond the effects of other retention strategy types. By contrast, Table 3-Model 2 demonstrates that when the retention rate was regressed on to all the binary indicator variables denoting whether the study did or did not utilise at least one strategy within that domain, only the use of follow-up/reminder strategies was independently associated with reduced retention rate (b = − 0.83; 95%CI [− 1.4 to − 0.27]; *p* < .01).

**Table 3:** Meta-analytic regression results between retention strategy themes and retention rate

	Estimate	CI (Lower - Upper)	*P*	I^2^
Model 1: Continuous total number of retention strategy types				99.86%
Barriers	0.17	0.03–0.32	0.02*	
Community	−0.03	− 0.18 - 0.11	0.63	
Follow-up/reminder	−0.15	−0.29 - -0.01	0.04*	
Tracing	0.11	−0.06 - 0.27	0.22	
Study duration	−0.04	−0.08 - 0.00	0.06	
Number of waves	0.00	−0.02 - 0.03	0.81	
Model 2: Binary usage of retention strategy types				99.84%
Barriers	0.35	−0.15 - 0.86	0.16	
Community	0.35	−0.14 - 0.83	0.16	
Follow-up/reminder	−0.83	−1.40 - -0.27	0.00**	
Tracing	0.11	−0.36 - 0.59	0.64	
Study duration	−0.03	−0.08 - 0.01	0.10	
Number of waves	0.01	−0.02 - 0.03	0.61	
Model 3: All individual strategies with p < 0.1				99.85%
Tracing - Locator form documentation	0.59	−0.44 - 1.62	0.26	
Follow-up - Reminder Phone call	−0.72	−1.20 - -0.25	0.00**	
Community - Thank you and birthday cards	0.44	−0.11 - 0.98	0.12	
Barriers - Site and home visits	0.42	−0.05 - 0.88	0.08	
Barriers - Consistency in research staff	0.39	−0.42 - 1.20	0.34	
Barriers - Alternative method of data collection	0.59	0.14–1.05	0.01**	
Study duration	−0.04	− 0.08 - − 0.00	0.05*	
Number of waves	-0.00	−0.03 - 0.02	0.89	

*p < .05

**p < .01

Finally, we investigated whether the associations between individual strategies and retention rate remained after controlling for other effective individual strategies in a single model (see Table 3 Model 3). A meta-regression model was created by entering only individual retention strategies that were associated with a retention rate at the *p* < .10 level (as discussed in [23, 24]). Six individual strategies were eligible: (i) offering alternative methods of data collection; (ii) consistency in the research staff; (iii) offering site and home visits; (iv) thank you and birthday cards; (v) phone call reminders; and (vi) the use of a locator form (i.e., alternate contacts). Offering participants alternative methods of data collection was associated with improved retention, whilst the use of phone call reminders was associated with reduced retention (b = 0.59; 95%CI [0.13 to 1.05]; *p =* 0.01; b = − 0.72; 95%CI [− 1.18 to − 0.25]; *p* < .01, respectively). No associations were found between retention rates and the remaining four individual strategies.

### Relationship between retention rate and emerging strategies

The final group-level analysis investigated the association between emerging retention strategies and retention rates. Within these 95 retention strategies, 44 emerging strategies were identified, including the application of social media and SMS to assist in tracing participants lost to follow-up, and the application of study websites and social media profiles for keeping participants up-to-date with the study’s news and events. Meta-regressions demonstrated that articles reporting a higher frequency of emerging retention strategies had higher retention, after controlling for study duration and number of waves (b = 0.08; 95%CI [0.01 to 0.16]; *p =* .03). Despite this, there was no difference in overall retention rates between those articles that did and did not report the use of emerging retention strategies (median retention using emerging strategies = 80.1%; median retention not using emerging strategies = 75.0%; b = 0.27, *p* = .27).

## Discussion

This study aimed to identify retention strategies employed in longitudinal cohort studies during the past decade, and to examine their effectiveness. We identified 143 longitudinal cohort studies that described retention strategies and outcomes, resulting in 95 different retention strategies. We then investigated whether study or participant characteristics moderated retention, the relationship between retention rate and retention strategy type, and whether new cohort retention strategies have emerged since previous reviews. In so doing, this study is the first meta-analysis of retention strategies conducted in longitudinal cohort studies. This research particularly complements the previous narrative review that investigated cohort retention strategies in longitudinal research [6], and the wider literature investigating participant retention strategies across health research designs (e.g., 4,16,17). Such research has important implications for maximising cohort retention and reducing research administration costs, which will subsequently improve the efficacy and quality of health research.

We first investigated how study or participant characteristics may influence cohort retention. Study characteristics included sample size, study duration, number of waves, and country development level - none of which were associated with retention rate. Participant characteristics included mean age at baseline, cohort type (clinical or non-clinical), and gender. We found that cohort studies with a higher proportion of male participants had lower retention rates than studies with a higher proportion of female participants; no associations were found for participants’ age or cohort type. While difficulties in retaining male participants are well-documented in previous research (e.g., 4,20,21), our study noted that cohorts with a higher proportion of male participants were also more likely to be clinical samples than cohorts with a higher proportion of female participants. In addition, cohorts with a higher proportion of male participants were also disproportionately focused on high-risk groups, such as substance use and men who have sex with men (e.g., the Bangkok Men who have Sex with Men Cohort Study (BMCS) [25] and the International Multicenter ADHD Genetics (IMAGE) study [26]). Thus, the difficulties in retention reported in this study and the wider literature could potentially be attributed to the differential impact of these clinical issues that affect men more than women. Researchers working with hard-to-retain populations, such as men in particular clinical groupings, may benefit from investigating what retention strategies work within their specific populations and settings beyond the core retention strategies identified in this review.

Second, we investigated the relationship between retention rate and retention strategies. We identified 95 different retention strategies, grouped thematically into four classes: *barrier-reduction, community-building, follow-up,* and *tracing*. Specific strategies associated with improved retention rates included the barrier-reduction strategy of offering alternative methods of data collection to participants (e.g., completing an interview over the phone or in person); and the tracing strategy of collecting detailed contact information from participants at baseline via a locator document. Further, weak evidence was found for one community-building and two further barrier-reduction strategies: (i) sending participants thank you, birthday or holiday cards; (ii) having consistent research team members, and; (iii) offering site and home visits for data collection.

Overall, barrier-reduction strategies emerged as the strongest predictor of improved retention. Barrier-reduction strategies may be particularly useful in longitudinal research given participants are likely to experience significant changes in their capacity to remain involved across the study’s duration (typically years). Follow-up/reminder strategies, such as incentives and reminders, were associated with significantly poorer retention. This result was surprising, given that the previous review investigating retention strategies in longitudinal cohort studies found the opposite, that use of these follow-up/reminder strategies resulted in improved retention rates [6]. The lack of support for follow-up/reminder strategies found in the current review could be due to a number of extraneous variables including: (i) timing: studies may have implemented this strategy after other retention efforts proved ineffective; (ii) participant burden: the studies using follow-up/reminder strategies may have involved a high data collection burden (e.g., long surveys); (iii) sampling: studies using follow-up/reminder strategies may be over-represented in studies of difficult-to-retain populations, such as men. However, these explanations are unlikely, given that follow-up/reminder strategies were identified in most of the cohorts included in this review (111 out of the 143 cohorts), and the cohorts employing follow-up/reminder strategies did not differ by research design (sample size: *t*_(141)_ = .67, *p* = .50; no. waves: *t*_(141)_ = −.43, *p* = .67) or participant characteristics (age: *t*_(141)_ = −.11, *p* = .91; gender: χ^2^_(2, *n* = 143)_ = .37, *p* = .85; HDI: χ^2^_(2, n = 143)_ = .01, *p* = .97). Differences were observed only for study duration (any *M*(SD) = 3.9(4.4); none (SD) = 5.8(6.4); *t*_(141)_ = 2.00, *p* = .05). Alternatively, participants may perhaps view follow-up/reminder strategies as the research team “badgering” them to complete assessments, thereby damaging rapport. This negative perspective of follow-up/reminder strategies may be further exacerbated if the research team has not implemented sufficient barrier-reduction strategies to help make it easier for participants to remain involved in the study. Future research could consider investigating participants’ perspectives of retention strategies in longitudinal cohort studies, ensuring that both active and inactive participants are included, to better understand the costs and benefits of different approaches.

Interestingly, the current study found that simply adding more cohort retention strategies did not result in higher retention rates. These results contradict the findings of Robinson et al. [17] and Davis et al. [4], who both found that the use of more retention strategies across multiple classes was associated with improved retention rates. However, neither study specifically examined participant retention in longitudinal cohort studies, and both synthesised their retention results using a narrative rather than meta-analytic approach. Given that the implementation of retention strategies can be costly in terms of both time and money, the overall number of strategies employed is important to evaluate. The interaction of quantity of retention strategies used and provision of flexibility needs to be better understood, given research protocols that accommodate the changing lives of participants should remain a key focus of retention efforts.

Finally, we examined whether studies utilising new or emerging retention strategies had improved retention compared with studies using established strategies. Of the 95 retention strategies described in the included articles, 44 were identified as an *emerging* retention strategy that had not yet been described in extant systematic reviews examining participant retention [4, 6, 16, 17]. Emerging strategies included using social media and SMS to assist in tracing participants lost to follow-up, and the use of study websites and social media profiles for keeping participants up-to-date with study news and events. Emerging retention strategies were endorsed by only a handful of studies, and the use of a single emerging strategy was not significantly associated with retention rate. However, we found that studies that employed more emerging retention strategies were associated with improved retention rates. Importantly, emerging strategies were identified across all four retention strategy domains (barrier-reduction, community-building, follow-up/reminder, and tracing), demonstrating that the association between emerging strategies and improvements in retention are due to the use of modern technology to help achieve core cohort engagement goals. Thus, we recommend that researchers continue to innovate their retention efforts, particularly where such strategies may reduce participant burden.

The current study has a number of limitations. First, the number of articles that focused on reporting retention strategies in detail was proportionally low compared to the number of articles that did not focus on reporting retention strategies. Although retention strategies were identified within 143 longitudinal cohorts, only 55 included cohort retention as a key focus area. Very few articles (*n* = 12) were identified that reported strategy-specific retention rates within the longitudinal cohort studies. The number of retention strategies reported by articles ranged from one to 32, with 35 of the 141 articles describing only one retention strategy. Longitudinal cohort studies should aim to publish protocol papers that delineate their cohort retention strategies, and ensure that the protocol is updated as retention efforts evolve.

Second, net retention rates were calculated by the difference between the first and last wave of data collection reported in the article. Where specified, ineligible participants (e.g., participants recruited after the first wave, or deceased participants) were excluded from the retention rate calculation. However, some articles did not provide detailed information on the eligibility of the sample at the final wave, and thus it is possible that the retention rates calculated for some studies may be slightly inaccurate. This limitation could be addressed by researchers providing details on the eligibility of their samples at each wave.

Third, high levels of heterogeneity were reported for most analyses in this study. This may best be explained by two factors. First, we expected to identify high heterogeneity given the diversity of research questions, methodologies, and cohorts reported across articles. Second, only a small number of studies were eligible for most meta-regressions in this paper, which reduces the precision of heterogeneity estimates [27]. This limitation could be addressed in future work, which could aim to investigate the effectiveness of different retention strategies within different subgroups.

Finally, by nature of synthesising retention results across different samples and settings, the current study is unable to disaggregate nuanced effects of various retention strategies across specific contexts and populations, given results are pooled across multiple studies. The current study did address this broadly by investigating the effects of study and sample characteristics on retention.

A final point to note is that available to researchers are a range of statistical or methodological approaches that can minimise potential biases introduced with attrition. Whilst beyond the scope of this paper, these approaches include formal statistical methods for addressing missingness due to attrition such as multiple imputation or full information maximum likelihood methods [28, 29]. Moreover, researchers may address attrition methodologically by using replacement sampling approaches that recruit new participants into a study to replace those who have dropped out, based on shared characteristics measured in the original sampling frame [30, 31]. All these methods provide useful avenues to address attrition once any employed retention strategies have been used to retain the largest proportion of the original sample as possible.

## Conclusions

Overall, this study has important implications for the retention efforts of longitudinal cohort studies. Combined, these results suggest that researchers need to be strategic in choosing how to invest their resources to better target participant retention, rather than simply increasing the number of strategies applied. Projects should invest both time and funding into matching retention strategies to the sample prior to implementation, including careful consideration of unintended burden for participants. Finally, given the high number of emerging retention strategies identified, longitudinal research methods clearly continue to evolve. Longitudinal cohort studies may benefit from open and regular protocol revision to incorporate new strategies, particularly where these strategies may offer greater flexibility to participants.

## Additional file

Additional file 1: **Table S1.** Terms used in the electronic search strategy, adjusted as required for each database. (DOCX 12 kb)

## Abbreviations

ADHD: Attention Deficit Hyperactivity Disorder
AIBL: Australian Imaging, Biomarkers and Lifestyle Flagship Study of Ageing
AIDS: Acquired Immune Deficiency Syndrome
ATAP: Australian Twin ADHD project
BGALS: Berkeley Girls with ADHD Longitudinal Study
BMCS: Bangkok Men who have Sex with Men Cohort Study
BT20: Birth to Twenty birth cohort
CAS(K): Knee clinical assessment study
CHS: Cardiovascular health study
CI: Confidence interval
CIRIS: Chlamydia Incidence and Re-infection Rates Study
CLHLS: Chinese Longitudinal Healthy Longevity Survey
COHRA2: Center for Oral Health Research in Appalachia 2 Study
DCHS: Drakenstein Child Health Study
EARTH: Education and Research Towards Health study
ECAGE: Study of Food Intake and Eating Behavior of Pregnant Women
EHL: Environments for Healthy Living
FAMAS: Florey Adelaide Male Ageing Study
FIOCRUZ: Fundação Oswaldo Cruz Rio de Janeiro
GGNET: G-GrippeNet Project
GROW: Geographic research on wellbeing study
HANDLS: Healthy Aging in Neighborhoods of Diversity across the Life Span study
HAS: Helsinki Aging Study
HDI: Human Development Index
Heart SCORE: Heart Strategies Concentrating on Risk Evaluation study
HIV: Human Immunodeficiency Virus infection
IMAGE: International Multicenter ADHD Genetics study
IMPAACT: International Maternal Pediatric Adolescent AIDS Clinical Trials
IPEC: Instituto de Pesquisa Clínica Evandro Chagas
IYDS: International Youth Development Study
JCS: Japan Children’s Study
LASA: Longitudinal Aging Study Amsterdam
LAW: Longitudinal Assessment of Women
M: Mean
MARI: Maternal Anxiety in Relation to Infant Development Study
MASALA: Mediators of Atherosclerosis in South Asians Living in America study
MIDUS: Midlife in the United States
MLS: Maternal Lifestyle Study
MTFS: Minnesota Twin Family Study
MUSP: Mater-University Study of Pregnancy
NECS: New England Centenarian Study
NEMESIS-2: Netherlands Mental Health Survey and Incidence Study
NESDA: Netherlands Study of Depression and Anxiety
NINFEA: Nascita e INFanzia gli Effetti dell’Ambiente cohort
NLLFS: US National Longitudinal Lesbian Family Study
NSHD: National Survey of Health and Development
OPPERA: Orofacial Pain: Prospective Evaluation and Risk Assessment Study
PRC: People’s Republic of China
PRISMA: Preferred Reporting Items for Systematic Reviews and Meta-Analyses
QLS: Quinte Longitudinal Study
RHIAA: Religion and Health in African Americans study
SD: Standard Deviation
SELF: Study of Environment, Lifestyle and Fibroids
SHIP: Study of Health in Pomerania
SMS: Short Message Services
SUPERB: Study of Use of Products and Exposure-Related Behavior
TEDDY: The Environmental Determinants of Diabetets in the Young study
TNTR: Tasmanian Neurotrauma Register
TRAILS: Tracking Adolescents’ Individual Lives Survey
UAB Study of Ageing: University of Alabama at Birmingham Study of Aging
UFS: Uterine Fibroid Study
USA: United States of America
USC RFAB: University of Southern California Study of Risk Factors for Antisocial Behavior
WCLS: Welfare Client Longitudinal Study
YAS: Youth Asset Study
