# Supplementary material for: Retention strategies in longitudinal cohort studies: a systematic review and meta-analysis
Source: BMC Med Res Methodol. 2018 Nov 26;18:151. doi: 10.1186/s12874-018-0586-7 (PMC6258319; doi:10.1186/s12874-018-0586-7)
Supplement: Supplementary file 1 — Table S1. Terms used in the electronic search strategy, adjusted as required for each database. (DOCX 12 kb) [file 12874_2018_586_MOESM1_ESM.docx]

# Supplementary Table 1

*Terms used in the electronic search strategy, adjusted as required for each database*

| Key search term | Adjacent terms |
| --- | --- |
| Attrition | Minimi*, prevent*, lessen*, decreas*, reduc* |
| Drop-out; drop-out*; drop*-out; dropout* | Minimi*, prevent*, lessen*, decreas*, reduc* |
| Follow-up; followup; follow-up stud*; followup stud* | Loss; lost |
| Withdrawal; withdrawal* | Minimi*, prevent*, lessen*, decreas*, reduc* |
| Retention | Increas*, encourag*, maximi*, promot*, improve*, strateg*, rate*, method*, technique*, difficult* |
| Questionnaire* adj3 response* | Increas*, encourag*, maximi*, promot*, improv*, strateg*, method*, rate* |
| Response* | Increas*, encourag*, maximi*, promot*, improv*, technique* |
| Retention rate* |  |
| Attrition rate* |  |
| Retain* | Difficult* |
| Longitudinal |  |
| Cohort |  |
| Prospect* |  |
| Patient dropout* |  |
